# The effect of uric acid and urinary sodium excretion on prehypertension: a nationwide population-based study

**DOI:** 10.1186/s12872-020-01535-9

**Published:** 2020-05-27

**Authors:** Shina Lee, Kyu Bok Choi, Seung-Jung Kim

**Affiliations:** Department of Internal Medicine, School of Medicine, Ewha Womans University, 1071, Anyangcheon-ro, Yangcheon-gu, Seoul, 158-710 Korea

**Keywords:** Prehypertension, Uric acid, Sodium

## Abstract

**Background:**

This study examined the effect of serum uric acid (SUA) level and urinary sodium excretion on blood pressure as well as their combined effect on prehypertension in a Korean population.

**Method:**

Data from the 7th Korea National Health and Nutrition Examination Survey for adults (≥ 19 years of age) were used. The participants were classified into two groups, normotension and prehypertension, according to the JNC-7 definition. Logistic regression was carried out and adjusted for traditionally regarded confounders of blood pressure. All analyses considered a complex sampling design. A multivariate analysis was performed on subgroups defined according to their SUA level and urinary sodium excretion.

**Results:**

The 4200 participants were divided into normotension (*n* = 2646) and prehypertension (*n* = 1554) groups. In the univariate analysis, patient age, male sex, concurrent comorbidity (diabetes mellitus, cardiovascular disease, stroke, dyslipidemia, and chronic kidney disease), uric acid, and urinary sodium excretion were associated with prehypertension. After adjusting for baseline covariates, both the SUA level and urinary sodium excretion were significant predictors of incident prehypertension (SUA, per 1 mg/dL increase, odds ratio [OR] 1.216, 95% confidence interval [95% CI] 1.131–1.309; urinary sodium excretion, per 1 g/day increase, OR 1.067, 95% CI 1.019–1.117). Additionally, simultaneously higher tertiles of SUA and urinary sodium excretion resulted in higher ORs for prehypertension.

**Conclusion:**

Increased SUA is a significant risk marker for the development of prehypertension in normotensives. Simultaneously high SUA and urinary sodium excretion amplified the effect on the development of prehypertension. Our findings suggest that lowering SUA levels and reducing sodium intake will contribute to preventing hypertension.

## Background

The prevalence of hypertension is increasing worldwide, posing a threat to public health. Moreover, hypertension is an absolute risk factor for cardiovascular morbidity and mortality, both of which continue to incur high health system costs [1, 2]. An effective measure to prevent incident hypertension is to reduce dietary sodium consumption, which in previous studies has been proven to lower blood pressure. The DASH-sodium (Dietary Approaches to Stop Hypertension) trial [3], a clinical feeding study designed to examine changes in blood pressure in response to three levels of sodium intake, demonstrated a significant positive relationship between sodium intake and blood pressure levels that was evident among participants with and without hypertension. A recent Cochrane review and meta-analysis of 34 randomized trials (3230 participants) showed that a mean change of 100 mmol/24 h reduction in urinary sodium reduced systolic and diastolic pressure by 5.4 mmHg and 2.8 mmHg, respectively, in hypertensive individuals, and by 2.4 mmHg and 1.0 mmHg, respectively, in normotensives [4].

The Seventh Report of the Joint National Committee (JNC-7) guideline defines prehypertension as a systolic blood pressure of 120–139 mmHg and/or a diastolic blood pressure of 80–90 mmHg [5]. Prehypertension is a powerful predictor for hypertension [5], and its risk factors include being overweight, excessive sodium intake, low physical activity, and excessive alcohol consumption, which are also risk factors for the developmet of hypertension [6, 7]. Investigations of the relationship between increased sodium intake and prehypertension identified endothelial dysfunction, oxidative stress, inflammation, insulin resistance, and peripheral resistance as causative factors [8, 9].

Uric acid, the metabolic end product of purine degradation, has been implicated as a potential risk factor and mediator of cardiovascular and renal diseases and their outcomes [10]. Furthermore, hyperuricemia has emerged as an important risk factor for both prehypertension and hypertension [11–13].

A recent study of a Chinese cohort examined the relationship between serum uric acid (SUA), urinary sodium excretion, and their interaction in the risk of prehypertension [13]. Although urinary sodium excretion was not significantly related to SUA, both were significantly associated with the risk of prehypertension, even after adjusting for multiple confounders. Additionally, a synergistic effect of SUA and urinary sodium excretion in the development of prehypertension was determined. However, the evidence whether SUA and urinary sodium excretion are also risk factors of prehypertension is scarce. Thus, in the present study, we used a nationwide database to examine whether SUA and urinary sodium excretion are risk factors for the development of prehypertension.

We hypothesized that SUA and excessive sodium intake would increase blood pressure, both individually and additively. We therefore examined the prevalence of prehypertension according to the SUA level and urinary sodium excretion, with the latter as a surrogate marker of salt intake. In addition, we performed a detailed analysis of the single and combined effects of SUA and urinary sodium excretion in elevating blood pressure.

## Methods

### Study design and participants

This study was conducted using data from 2016 to 2018, obtained from the Korean National Health and Nutrition Examination Survey (KNHANES) VII. KNHANES is a nationwide survey that has been conducted by the Korea Centers for Disease Control and Prevention since 1998. The newest published version was used in our study. The unbiased cross-sectional estimates reported in KNHANES are achieved with a complex multi-stage probability sample design based on age, sex, and geographic area to identify participants in the general Korean population. The survey is composed of a self-reported questionnaire addressing nutritional and heath related issues as well as health-related measurements, with the latter obtained by trained staff. Thus, the database is representative of the health and nutritional status, as well as health behaviors of the entire Korean population.

Of the 8127 Korean participants, the study population consisted of 6518 people aged 19 years or older. The exclusion criteria were as follows: individuals under the age of 19 years and those lacking data for the determination of blood pressure, SUA, and urinary sodium excretion. The study protocol was approved by the Institutional Review Board of Ewha (IRB no. EUMC 2020–01-010).

### Measurements and definitions of hypertension, prehypertension, diabetes mellitus, dyslipidemia, chronic kidney disease, and hyperuricemia

For the purposes of KNHANES, well-trained nurses measured blood pressure using a standard mercury sphygmomanometer (Baumanometer® wall unit 33, Baum Co., Inc., Copiague, NY, USA) with the participants in a sitting position after they had rested for at least 10 min. For all participants, blood pressure was measured on three separate occasions at 5-min intervals in a quiet setting. The mean of the last two measurements was adopted for the data analysis.

Based on the JNC-7 criteria, hypertension was defined in an individual taking anti-hypertensive medication or with a systolic blood pressure ≥ 140 mmHg or a diastolic blood pressure ≥ 90 mmHg [14]. Prehypertension was defined as a systolic blood pressure of 120–139 mmHg or a diastolic blood pressure of 80–89 mmHg. Normotension was thus defined as a systolic blood pressure < 120 mmHg and a diastolic blood pressure < 80 mmHg.

Diabetes mellitus (DM) was defined in individuals on medication for DM or with a HbA1c level ≥ 6.5%, according to the International Expert Committee [15]. Dyslipidemia, stroke, cardiovascular disease (CVD), and chronic kidney disease (CKD) were defined based on current disease status, treated with medication or not. CVD included angina or myocardial infarction as self-reported in the questionnaire.

### Biochemical analyses

Blood specimens were collected by peripheral venous puncture after the participants had fasted overnight (12-h fast). Biochemical analyses were performed centrally. Serum levels of glucose, total cholesterol, triglyceride, low-density lipoprotein cholesterol, serum creatinine, urine creatinine, and blood urea nitrogen (BUN) were measured using an automatic biochemical analyzer (model 7600–210, Hitachi, Tokyo, Japan), as was hemoglobin (XN-9000; Sysmex Corp., Kobe, Japan). High-sensitivity C-reactive protein (hs-CRP) was measured by immunoturbidimetry using a Cobas device (Roche, Mannheim, Germany). SUA was measured using the calorimetry (uricase) method and an automatic analyzer (Hitachi 7600–210). The estimated glomerular filtration rate (eGFR) was calculated using the Modification of Diet in Renal Disease equation.

Urinary sodium was measured in morning fasting midstream urine samples using an automatic analyzer (Hitachi 7600–210). The Tanaka equation, which has been validated in the Korean population [16], was applied to calculate 24-h urinary sodium excretion in spot urine samples.

### Statistical analysis

Factors related to prehypertension were analyzed using SPSS software (ver. 18.0 for Windows; SPSS, Chicago, IL, USA). The multivariate statistical analysis was performed using a weighted approach, according to the Korea Centers for Disease Control and Prevention’s guidelines for the use of primitive data. A complex sample plan file was designed to apply k strata, primary sample units, and proper usage of sampling weight values. The data are expressed as the absolute number and estimated percentage or mean ± standard error. The χ2 test was used to compare the comorbidity prevalence between the normotension and prehypertension groups. The relationship between blood pressure and each variable was investigated using a linear regression analysis, correcting for the confounding factors of age, sex, other diseases traditionally regarded as confounders for blood pressure, SUA, and urinary sodium excretion. Additionally, a multivariate logistic regression analysis was conducted to investigate the single and joined effects of SUA level and urinary sodium excretion in the development of prehypertension from normotension. Furthermore, To establish whether there was a likely causal relationship, rather than simply associative, stratified interaction terms were assessed and presented in the manner suggested by Knol and van Der Weele [17]. A *p*-value < 0.05 was considered to indicate statistical significance.

## Result

### Baseline characteristics of the study population

Of the 6518 individuals ≥19 years of age who were eligible for the study, 2318 were excluded from participation due to hypertension (*n* = 1978) and missing data (blood pressure, blood chemistry, urinary biochemistry; *n* = 340). Thus, the study population comprised the 4200 individuals who met the inclusion criteria. Of these, 2646 (66.2%) were assigned to the normotension arm and 1554 (33.8%) to the prehypertension arm, according to the JNC-7 classification of blood pressure. Table 1 shows the baseline characteristics of the two groups. The participants ranged in age from 19 to 80 years (mean = 47.3 ± 0.45 years), with a mean of 46.8 ± 0.64 years in the prehypertension group and 40.9 ± 0.38 years in the normotension group (*p* <  0.001). Nearly half of the normotensive participants were male (49.7 ± 0.7%), with a lower percentage in the prehypertension arm (39.4 ± 1.0%, p <  0.001).

**Table 1:** Baseline characteristics of participants categorized by blood pressure status. Data are presented as mean ± SE or weighted percentage (%) ± SE

Characteristics	Total (*n* = 4200)	Normotension (*n* = 2646, 66.2%)	Prehypertension (*n* = 1554, 33.8%)	*P* value
Age (years)	43.0 ± 0.4	40.9 ± 0.4	46.9 ± 0.6	< 0.001
Male (%)	47.3 ± 0.9	49.7 ± 0.7	39.4 ± 1.0	< 0.001
Body mass index (Kg/m^2^)	23.4 ± 0.1	22.8 ± 0.1	24.5 ± 0.1	< 0.001
Current smoking (%)	51.9 ± 1.6	53.7 ± 1.9	49.6 ± 2.3	< 0.001
Systolic blood pressure (mmHg)	111.8 ± 0.3	105.7 ± 0.2	122.7 ± 0.3	< 0.001
Diastolic blood pressure (mmHg)	73.6 ± 0.2	69.7 ± 0.2	80.7 ± 0.2	< 0.001
Comorbidity				
Diabetes mellitus (%)	5.3 ± 0.4	4.4 ± 0.4	7.1 ± 0.8	< 0.001
Cardiovascular disease (%)	1.1 ± 0.2	0.8 ± 0.1	1.7 ± 0.4	< 0.001
Stroke (%)	0.7 ± 0.1	0.5 ± 0.1	1.1 ± 0.3	< 0.001
Dyslipidemia (%)	9.8 ± 0.6	8.0 ± 0.6	13.0 ± 1.0	< 0.001
Chronic kidney disease (%)	0.11 ± 0.05	0.09 ± 0.05	0.13 ± 0.08	< 0.001
White blood cell (μL^− 1^)	6.13 ± 0.04	6.01 ± 0.04	6.34 ± 0.06	< 0.001
Hemoglobin (g/dL)	14.19 ± 0.33	13.89 ± 0.04	14.75 ± 0.05	< 0.001
Fasting glucose (mg/dL)	96.0 ± 0.4	94.1 ± 0.4	99.5 ± 0.7	< 0.001
Blood urea nitrogen (mg/dL)	13.5 ± 0.1	13.2 ± 0.1	14.0 ± 0.1	< 0.001
Serum creatinine (mg/dL)	0.82 ± 0.004	0.79 ± 0.004	0.86 ± 0.006	< 0.001
eGFR (mL/min/1.73m^2^)	97.1 ± 0.4	103.5 ± 0.4	96.5 ± 0.5	< 0.001
Total cholesterol (mg/dL)	194.6 ± 0.7	190.5 ± 0.8	202.1 ± 1.1	< 0.001
Triglycerides (mg/dL)	126.9 ± 1.9	111.4 ± 1.9	155.1 ± 4.4	< 0.001
LDL-cholesterol (mg/dL)	125.5 ± 2.0	124.4 ± 2.9	126.3 ± 2.5	0.60
HDL-cholesterol (mg/dL)	52.1 ± 0.3	52.9 ± 0.3	50.6 ± 0.4	< 0.001
hs-CRP (mg/dL)	1.04 ± 0.03	0.97 ± 0.03	1.17 ± 0.05	< 0.001
Serum uric acid (mg/dL)	5.09 ± 0.03	4.91 ± 0.04	5.43 ± 0.05	< 0.001
Urinary sodium excretion (g/day)	7.54 ± 0.04	7.21 ± 0.05	7.72 ± 0.06	< 0.001

*SE* Standard error, *LDL* Low-density lipoprotein, *HDL* High-density lipoprotein, *GFR* Estimated glomerular filtration rate, *hs-CRP* High sensitivity C reactive protein

In the prehypertension groups, mean body mass index (BMI) was higher than in the normotension group, and the prevalences of DM, CVD, stroke, dyslipidemia, and CKD were higher. Mean hemoglobin level, white blood cell count, fasting glucose, BUN, serum creatinine, and hs-CRP concentrations were also significantly higher in the prehypertension than in the normotension group. The eGFR was significantly lower in the prehypertension group than in the normotension group. There was no significant difference in the smoking status of the two groups. Total cholesterol and triglyceride were higher in the prehypertension group, and the high-density lipoprotein (HDL) level was significantly lower in the prehypertensin. The difference in low-density lipoprotein (LDL) levels of the two groups was not significant.

The SUA level was significantly higher in the prehypertension than in the normotension group (5.43 ± 0.05 vs. 4.91 ± 0.04 mg/dL, *p* < 0.001), as was the estimated 24-h sodium excretion (7.21 ± 0.05 and 7.72 ± 0.06 g/day, respectively, p < 0.001).

### Odds ratios of uric acid and urinary sodium excretion for prehypertension

Logistic regression was conducted to evaluate the effects of SUA and urinary sodium excretion on blood pressure. Table 2 shows the crude odds ratios (ORs) for prehypertension vs. normotension. Age, male sex, BMI, DM, CVD, stoke, dyslipidemia, and CKD were associated with prehypertension. White blood cell count, hemoglobin, fasting glucose, serum creatinine, cholesterol, triglyceride, and hs-CRP were risk factors for prehypertension whereas the relationship between LDL and prehypertension was not significant. In the crude model, SUA and urinary sodium excretion were significantly associated with prehypertension (SUA per 1 mg/dL: OR 1.334, 95% CI: 1.265–1.407; urinary sodium excretion per 1 g/day: OR 1.140, 95% CI: 1.095–1.187, *p* < 0.001).

**Table 2:** Associations between various characteristics and prehypertension in crude model using logistic regression analysis

Characteristics	OR	95% CI	P value
Age (years)	1.027	1.022–1.033	< 0.001
Male	2.462	2.141–2.832	< 0.001
Body mass index (Kg/m^2^)	1.157	1.126–1.189	< 0.001
Current smoking	0.849	0.679–1.062	0.151
Systolic blood pressure (mmHg)	1.393	1.358–1.429	< 0.001
Diastolic blood pressure (mmHg)	1.379	1.339–1.420	< 0.001
Comorbidity			
Diabetes mellitus	1.662	1.214–2.277	< 0.001
Cardiovascular disease	2.129	1.160–3.908	< 0.001
Stroke	2.099	1.052–4.192	< 0.001
Dyslipidemia	1.721	1.364–2.172	< 0.001
Chronic kidney disease	1.391	0.232–8.351	0.716
White blood cell (μL^−1^)	1.123	1.079–1.168	< 0.001
Hemoglobin (g/dL)	1.418	1.344–1.495	< 0.001
Fasting glucose (mg/dL)	1.017	1.011–1.023	< 0.001
Blood urea nitrogen (mg/dL)	1.057	1.035–1.080	< 0.001
Serum creatinine (mg/dL)	1.247	1.170–1.329	< 0.001
eGFR (mL/min/1.73 m2)	0.970	0.966–0.975	< 0.001
Total cholesterol (mg/dL)	1.009	1.007–1.011	< 0.001
Triglycerides (mg/dL)	1.005	1.003–1.006	< 0.001
LDL-cholesterol (mg/dL)	1.002	0.996–1.007	0.603
HDL-cholesterol (mg/dL)	0.984	0.978–0.990	< 0.001
hs-CRP (mg/dL)	1.083	1.040–1.128	< 0.001
Serum uric acid (mg/dL)	1.334	1.265–1.407	< 0.001
Urinary sodium excretion (g/day)	1.140	1.095–1.187	< 0.001

*OR* odds ratio, *CI* confidence interval, *LDL* low-density lipoprotein, *HDL* high-density lipoprotein, *eGFR* estimated glomerular filtration rate, *hs-CRP* high sensitivity C reactive protein

Multivariable logistic regression analyses were then conducted and the ORs for developing prehypertension from normotension were calculated after adjustment for age, sex, dyslipidemia, stroke, CVD, CKD, DM, hs-CRP, SUA, and urinary sodium excretion, which were significant confounders in the crude model (Table 3). In model 1 (adjusted for age and sex), model 2 (additionally adjusted for disease status, including dyslipidemia, stroke, CVD, CKD, and DM), and model 3 (additionally adjusted for hs-CRP), SUA and sodium excretion remained significant predictors of prehypertension.

**Table 3:** Association of serum uric acid and urinary sodium excretion with prehypertension by multiple logistic regression. Model 1: age and sex were adjusted. Model 2: age, sex and comorbidties including diabetes mellitus, cardiovascular disease, stroke, dyslipidemia, and chronic kidney disease were adjusted. Model 3: age, sex, comorbidities, and high sensitivity reactive protein were adjusted

	Model 1			Model 2			Model 3		
	OR	95% CI	P value	OR	95% CI	P value	OR	95% CI	P value
Serum uric acid, per 1 mg/dL increased	1.239	1.158–1.326	< 0.001	1.230	1.143–1.324	< 0.001	1.216	1.131–1.309	< 0.001
Urinary sodium excretion, per 1 g/day increased	1.077	1.032–1.125	< 0.001	1.064	1.018–1.112	0.006	1.067	1.019–1.117	0.006

*OR* odds ratio, *CI* confidence interval

### Odds ratios of SUA and urinary sodium excretion tertiles for prehypertension

**Fig. 1:**
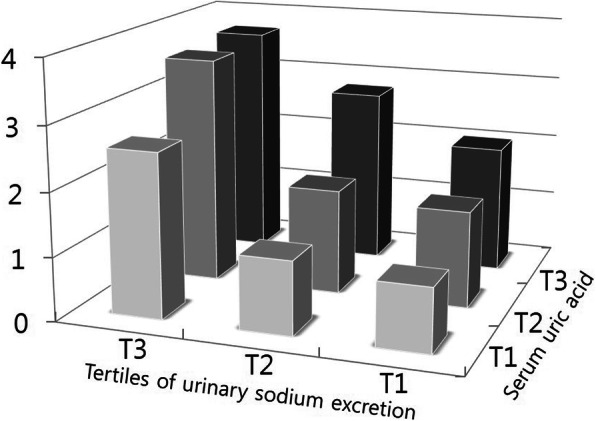
Combined effect of serum uric acid and urinary sodium excretion on the risk of prehypertension. The height of each bar represents the OR for each group. ORs are compared with a common reference group (the lowest tertile of urinary sodium excretion and either the lowest tertile of serum uric acid)

To address the effect of SUA and urinary sodium excretion on incident prehypertension, the participants were classified according to their SUA level and urinary sodium excretion to obtain the ORs. The tertiles of SUA were as follows: tertile 1, < 4 g/dL; tertile 2, 4–5 g/dL, and tertile 3, 6–13 g/dL; those of urinary sodium excretion were as follows: tertile 1, 2.12–6.44 g/day; tertile 2, 6.44–8.24 g/day, and tertile 3, 8.24–21.32 g/day. Table 4 shows the OR of each tertile of SUA and urinary sodium excretion for prehypertension vs. normotension. In the analysis of the tertiles of both parameters, the lowest tertile (tertile 1) served as the reference (OR of 1.00). In a crude model, the OR of prehypertension for the risk of prehypertension increased across SUA tertiles 2 and 3, to 1.250 (95% CI: 1.020–1.533) and 2.328 (95% CI: 1.958–2.768), respectively (p for trend < 0.001). The ORs of urinary sodium excretion also increased significantly, to 1.418 (95% CI, 1.169–1.719) and 1.820 (95% CI, 1.502–2.204), respectively (p for trend < 0.001). Figure 1 illustrates the increased ORs across the increasing both SUA and urinary sodium excretion tertiles on the risk of prehypertension.

**Table 4:** Association between each tertile of serum uric acid and urinary sodium excretion by multiple logistic regression. Model 1: age and sex were adjusted. Model 2: age, sex, and comorbidities including diabetes mellitus, cardiovascular disease, stroke, dyslipidemia, and chronic kidney disease were adjusted. Model 3: age, sex, comorbidities, and high sensitivity reactive protein were adjusted

		Crude		Model 1		Model 2		Model 3	
		OR	95% CI	OR	95% CI	OR	95% CI	OR	95% CI
Serum uric acid	Tertile 1	Reference		Reference		Reference		Reference	
	Tertile 2	1.250	1.020–1.533	1.077	0.872–1.332	1.048	0.840–1.309	1.025	0.816–1.287
	Tertile 3	2.328	1.958–2.768	1.746	1.410–2.162	1.702	1.356–2.135	1.597	1.267–2.013
	P for trend	< 0.001		< 0.001		< 0.001		< 0.001	
Urinary sodium excretion	Tertile 1	Reference		Reference		Reference		Reference	
	Tertile 2	1.418	1.169–1.719	1.200	0.984–1.463	1.191	0.959–1.479	1.167	0.939–1.451
	Tertile 3	1.820	1.502–2.204	1.370	1.122–1.673	1.299	1.052–1.603	1.317	1.060–1.637
	P for trend	< 0.001		< 0.001		0.053		< 0.05	

*OR* odds ratio, *CI* confidence interval

In the model adjusting for age and sex (model 1), the OR of SUA for prehypertension increased across the increasing SUA tertiles, and the OR of the highest tertile was significant (OR 1.746, 95% CI: 1.410–2.162). After additional adjustment for DM, CVD, stroke, dyslipidemia, and CKD (model 2), the OR for prehypertension of the highest SUA tertile remained significant (OR 1.702, 95% CI: 1.356–2.135). With the additional adjustment of hs-CRP (model 3), the highest SUA tertile was also significant for prehypertension (OR 1.597, 95% CI: 1.267–2.013). Similarly, in the model based on urinary sodium excretion and adjusted for age and sex (model 1), the OR increased as urinary sodium excretion increased and the OR of the highest tertile was significant (OR 1.370, 95% CI: 1.122–1.673). After stepwise adjustment for disease status and hs-CRP (models 2 and 3, respectively), the highest tertile of urinary sodium excretion was a significant predictor of prehypertension.

### Combined effect of SUA and urinary sodium excretion for prehypertension

We then examined whether SUA in combination with urinary sodium excretion had an amplified effect on the development of prehypertension in previously normotensive individuals. The study participants were assigned to one of nine subgroups according to their SUA and urinary sodium excretion tertiles (Table 5). Tertile 1 of SUA and urinary sodium excretion combined was set as the reference and a logistic regression analysis was performed to estimate the ORs for prehypertension vs. normotension. The ORs increased as the tertiles of both SUA and urinary excretion increased. The results showed that, compared to the reference tertile (tertile 1: OR 1.00), the OR of prehypertension was three times higher for the highest combined SUA and urinary sodium excretion tertiles (tertile 3), with an OR of 3.679 (95% CI: 2.608–5.191). Within the strata of urinary sodium excretion tertiles, tertile 3 of SUA had significant ORs (OR, 95% CI for T3 of uric acid within strata of urinary sodium excretion tertiles: 2.582, 1.805–3.693 for tertile 1; 2.355, 1.764–3.144 for tertile 2; 1.826, 1.357–2.459 for tertile 3). Within the strata of SUA tertiles, tertile 3 of urinary sodium excretion had a higher risk for prehypertension than those of tertile 2. In the interaction analysis, a significant interaction was found on an additive scale between tertile 3 of urinary excretion sodium and tertile 2 of serum uric acid (Relative excess risk due to interaction, 95% Confidence Interval: 0.293, 0.036–0.549). Statistically, estimated interactions on multiplicative scales showed non-significance between each of the tertiles of SUA and urinary sodium excretion.

**Table 5:** Combined effect on serum uric acid and urinary sodium excretion on the risk of prehypertension from normotension

		Urinary sodium excretion									
		Tertile 1		Tertile 2		Tertile 3		ORs (95% CI) for T2 of urinary sodium excretion within strata of serum uric acid tertiles		ORs (95% CI) for T3 of urinary sodium excretion within strata of serum uric acid tertiles	
		OR	95% CI	OR	95% CI	OR	95% CI				
Serum uric acid	Tertile 1	Reference		1.520	1.053–2.195	2.015	1.427–2.844	1.520	1.053–2.195	2.015	1.427–2.844
	Tertile 2	1.155	0.805–1.657	1.654	1.157–2.364	2.771	1.931–3.976	1.432	0.989–2.074	2.400	1.672–3.443
	Tertile 3	2.582	1.805–3.693	3.580	2.570–4.988	3.679	2.608–5.191	1.386	1.016–1.892	1.425	1.031–1.970
ORs (95% CI) for T2 of uric acid within strata of Urinary sodium excretion tertiels		1.155	0.805–1.657	1.088	0.782–1.513	1.375	0.978–1.934				
ORs (95% CI) for T3 of uric acid within strata of Urinary sodium excretion tertiels		2.582	1.805–3.693	2.355	1.764–3.144	1.826	1.357–2.459				
Measure of interaction on additive scale: RERI (95% CI)						0.293^a^	0.036–0.549				
				0.810^b^	−0.450 - 2.06	1.856^c^	−1.078 - 4.790				
Measure of interaction on multiplicative scale: Ratio of ORs (95% CI)						1.191^d^	0.740–1.918				
				0.912^e^	0.571–1.458	0.841^f^	0.662–1.069				

^a,c^Measure of interaction for tertile 3 urinary sodium excretion and tertile 2 serum uric acid; ^b,e^Measure of interaction for tertile 2 urinary sodium excretion and tertile 3 serum uric acid; ^c,f^Measure of interaction for tertile 3 urinary sodium excretion and tertile 3 serum uric acid

*OR* odds ratio, *CI* confidence interval, *RERI* relative excess risk due to interaction

## Discussion

Prehypertension is a well known precursor of hypertension and is associated with morbidity and mortality from cardiovascular causes [5, 18]. Given the high incidence of prehypertension (30–50%) in many countries, studies of measures aimed at reducing the development of incident hypertension have been conducted [19, 20], such as the above-mentioned DASH trial. In a previous large-scale, long-term, community-based randomized controlled trial, the authors found that a moderate reduction of dietary sodium intake resulted in an additional 4.3 mmHg reduction in systolic blood pressure among older persons with well-controlled hypertension [21]. Meta-analyses and Cochrane reviews have confirmed the efficacy of reduced sodium intake in lowering blood pressure [4, 22, 23]. However, while these studies clearly determined that a lower salt intake lowered blood pressure in people with or without hypertension, few studies have specifically examined the effect of a reduced salt intake in the population with prehypertension.

Many epidemiologic studies have demonstrated a strong association between SUA levels and hypertension. A 5-year Japanese cohort study showed that SUA was an important risk factor for developing hypertension from prehypertension (OR 1.149, 95% CI: 1.066–1.238), even after multivariable adjustments [24]. In the Framingham Heart Study, each 1.3 mg/dL increase in SUA was associated with an OR of 1.17 (95% CI: 1.02–1.33) for developing hypertension [25]. A recent systemic review and meta-analysis revealed an association between a 1.0 mg/dL (60 μmol/L) increase in SUA and an increased risk of incident hypertension of 13% (95% CI: 1.06–1.20) [26]. Among the few studies investigating the association between SUA and incident prehypertension, a cross-sectional study of a nationally representative sample drawn from the US adult population demonstrated a positive association [27]. Moreover, in a subgroup analysis within the same study, a distinct OR according to race-ethnicity was determined for this association. In Asia, a Chinese prospective cohort study in which participants were recruited from a single center, SUA was identified as an independent predictor of the development of prehypertension, with increasing ORs across SUA quintiles [11]. A cross-sectional study from Bangladesh, in which the study population consisted of the staff and young students of two educational facilities, also found a relationship between the SUA level and prehypertension [12]. However, before a consensus can be reached, data from various countries and races are needed. Our study is the first study to evaluate the relationship between SUA and urinary sodium excretion in the development of prehypertension in Korean adults. The present study of a large Korean population determined a positive association between SUA and prehypertension and the combined effect on this association of an increasing urinary sodium excretion. Of note, since the interaction analyses did not reach statistical significance from multiplicativity, combined effect is more appropriate expression rather than synergistic effect.

The mechanism underlying the combined effect of sodium intake and SUA on prehypertension is unclear, but an experimental animal study suggested that hyperuricemia induces renal afferent arteriolopathy, resulting in a decreased arteriolar lumen diameter and an increased media to lumen ratio, changes that are also commonly observed in hypertension [28]. In an experimental study in which rats were randomized to a low- or high-salt diet, an increase in blood pressure in the high-salt group was observed only in previously hyperuricemic rats, with hyperuricemia considered indicative of high salt sensitivity. In another animal study, hyperuricemia induced high blood pressure via the upregulation of ENaC (epithelial sodium channels on the apical membrane of epithelial cells in the distal kidney tubules) [29]. A cross-sectional study in humans demonstrated the potential contributions of a high SUA level, a high salt intake, and their synergistic interaction in the development of hypertension. The authors postulated that a high salt intake enhanced the associations of the SUA level with hypertension and cardiovascular risk [30]. A prospective, large, population-based study of the effect of high sodium intake showed that for each 1-g increase in sodium intake the hazard ratio for developing hypertension increased significantly for the highest vs. lowest SUA tertile (OR 1.09, 95% CI: 1.02–1.16 and OR 0.98, 95% CI: 0.89–1.08, respectively) [31]. These findings are in agreement with those of the present study, in that the ORs for developing prehypertension determined for the highest tertiles of SUA and urinary sodium excretion increased significantly, especially in the adjusted models (Table 4). Taken together, the results demonstrate the amplified effect of a high SUA and high sodium intake on an increase in blood pressure.

Our finding of an association between increased SUA and the development of prehypertension is largely in agreement with previous studies. However, differences between our results and those of other studies were determined with respect to dyslipidemia. Dyslipidemia, defined as an altered ratio of high total cholesterol level and abnormal LDL or triglyceride levels, is usually associated with increased blood pressure [32, 33]. A cross-sectional study documented an association of the lipid profile with prehypertension status, in which, after multivariate adjustment, triglyceride was a significant determinant of prehypertension in women (OR 1.003, 95% CI: 1.000–1.005) [34]. In the present study, triglyceride and total cholesterol were positively associated with the development of prehypertension, but LDL cholesterol was not. However, current dyslipidemia remained a significant covariate even after adjusting for multiple confounders. It is likely that dyslipidemia plays an indirect role in the pathogenesis of increasing blood pressure, one that is mediated by endothelial dysfunction, as a high LDL cholesterol level inhibits the endothelium-dependent vasodilator response to acetylcholine [35, 36] and oxidized, but not native LDL, induces atherosclerosis [37].

Thus, our study suggests that targeting normotensive individuals who have a high SUA may be effective in preventing prehypertension, at least in the Korean population. This effect of hyperuricemia was shown to be amplified by a high-salt diet.

Our study had several limitations. First, although it was conducted based upon a nationwide population, participation in the database is voluntary, such that the participants may have been healthier than those who refused participation, which may have introduced selection bias. Also, the cross-sectional design of this study may have altered the cause-effect relationship between the SUA level and increased blood pressure. Second, we used the Tanaka formula and a spot urine specimen [38] to estimate 24-h urine sodium excretion, which may have been less accurate than measuring sodium excretion in a true 24-h urine sample. The accuracy of the estimated 24-h sodium excretion based on a spot urine sample mostly depends on the accuracy of the estimated 24-h creatinine excretion, which is based on sex, weight, and height but is also potentially influenced by diet and lean body mass [39, 40]. However, given the high burden on participants of 24-h urine collection, the time demands and costs of a large-scale survey and the practical issues related to an outpatient setting, a spot urine sample is a reasonable choice for estimating 24-h sodium excretion. Moreover, a Korean working group evaluated the reliability of the Tanaka method using spot urine samples to estimate 24-h urine sodium excretion [16] and found that it was not inferior to the Intersalt equation using casual spot urine. Third, KHANES did not include detailed information about current medication use. This lack of information on drugs, including those affecting urinary sodium excretion directly, such as nonsteroidal anti-inflammatory drugs, diuretics, and urate-lowering agents (including allopurinol and febuxostat), may have led to results that disagreed with those of previous studies.

Nonetheless, our study suggests that intensive modification of sodium intake together with a reduction of SUA is needed to prevent prehypertension in normotensive individuals. A multi-center, prospective trial will be essential to understand the benefits that can be achieved from the treatment of a high SUA level in preventing prehypertension.

## Conclusion

The present study demonstrated that an increased sodium intake together with a high SUA level increased the risk of incident prehypertension in the Korean population. In addition, normotensive individuals with a concurrent elevated SUA level and high sodium intake had a higher risk of developing prehypertension.

## Data Availability

The datasets analysed during the current study are available in the Korea Centers for Disease Control and Prevention repository, [https://knhanes.cdc.go.kr/knhanes/sub03 /sub03_02_02.do].

## Abbreviations

SUA: Serum uric acid
DM: Diabetes mellitus
CVD: Cardiovascular disease
CKD: Chronic kidney disease
BUN: Blood urea nitrogen
hs-CRP: High-sensitivity C-reactive protein
eGFR: Estimated glomerular filtration rate
HDL: High-density lipoprotein
LDL: Low-density lipoprotein
OR: Odds ratios
CI: Confidence intervals
BMI: Body mass index
